# Therapeutic Targeting of Protein Tyrosine Phosphatases from *Mycobacterium tuberculosis*

**DOI:** 10.3390/microorganisms9010014

**Published:** 2020-12-23

**Authors:** Kasi Viswanatharaju Ruddraraju, Devesh Aggarwal, Zhong-Yin Zhang

**Affiliations:** 1 Department of Medicinal Chemistry and Molecular Pharmacology, Purdue University, West Lafayette, IN 47907, USA; kruddrar@purdue.edu (K.V.R.); aggarw70@purdue.edu (D.A.); 2 Department of Chemistry, Purdue University, West Lafayette, IN 47907, USA; 3 Institute for Drug Discovery, Purdue University, West Lafayette, IN 47907, USA

**Keywords:** protein tyrosine phosphatases (PTPs), tuberculosis (TB), *Mycobacterium tuberculosis* (Mtb), signal transduction, virulence factors, pathogenic microorganisms, PTP inhibitors, salicylic acid derivatives, oxamic acids

## Abstract

Tuberculosis (TB) is an airborne infectious disease caused by *Mycobacterium tuberculosis* (Mtb). According to the World Health Organization, an estimated 10 million people developed TB in 2018. The occurrence of drug-resistant TB demands therapeutic agents with novel mechanisms of action. Antivirulence is an alternative strategy that targets bacterial virulence factors instead of central growth pathways to treat disease. *Mycobacterium* protein tyrosine phosphatases, mPTPA and mPTPB, are secreted by Mtb into the cytoplasm of macrophages and are required for survival and growth of infection within the host. Here we present recent advances in understanding the roles of mPTPA and mPTPB in the pathogenesis of TB. We also focus on potent, selective, and well-characterized small molecule inhibitors reported in the last decade for mPTPA and mPTPB.

## 1. Introduction

Tuberculosis (TB) is an airborne infectious disease caused by the intracellular pathogen *Mycobacterium tuberculosis* (Mtb). TB is one of the top 10 causes of human death worldwide and the leading cause of death from a single infectious agent. According to the World Health Organization (WHO, 2018 tuberculosis report), an estimated 10 million people developed TB, and over 1.2 million people succumbed to it in 2018. It is also estimated that about a quarter of the global population has latent TB. Current TB treatment is based on combination chemotherapy and requires 6 to 9 months of treatment. However, due to increasing drug resistance, antibiotics are losing efficacy in treating TB. Inadequate diagnosis, lack of compliance from patients, irregular drug supply, poor bioavailability of drugs, and mutation/gene transfer are some of the factors contributing to drug resistance [1]. According to the WHO, there are currently 50 antibiotics in the clinical pipeline [2]. However, this clinical pipeline is insufficient to tackle the problem of increasing antibiotic resistance. Since July 2017, eight new antibiotics have been approved, but many have limited clinical benefits compared to the existing treatments. The occurrence of multidrug-resistant and extensively drug-resistant tuberculosis demands the development of therapeutic agents with novel mechanisms of action [3]. Since early interactions between Mtb and the host innate immune system play essential roles in the establishment of TB infection and disease development, alternative therapeutic approaches such as antivirulence strategies are gaining interest among researchers in the development of more effective TB therapies and in combating antibiotic resistance.

**Figure 1:**
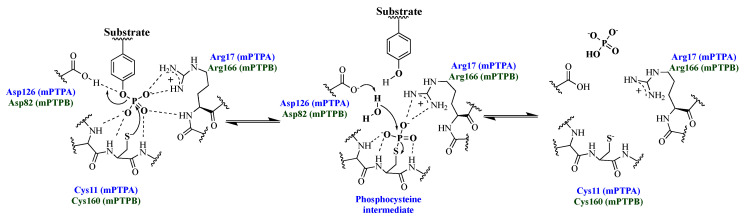
The dephosphorylation mechanism of protein tyrosine phosphatases with mPTPA and mPTPB as examples. The residues are labeled in blue for mPTPA and in green for mPTPB. The dianionic phosphotyrosine (pTyr) binds to the positively charged active site of PTPs. The active site cysteine thiol (Cys11 in mPTPA, Cys160 in mPTPB) possesses a pKa of ~5 and is predominantly in the thiolate form at neutral pH. When binding to pTyr substrates, aspartic acid, acting as a general acid (Asp126 in mPTPA, Asp82 in mPTPB), moves closer to the active site to neutralize the leaving group and facilitates nucleophilic attack by cysteine at the phosphorus atom of the substrate. The dephosphorylated substrate leaves the active site, giving a water molecule an opportunity to attack at the phosphocysteine intermediate to regenerate the cysteine thiolate.

**Figure 2:**
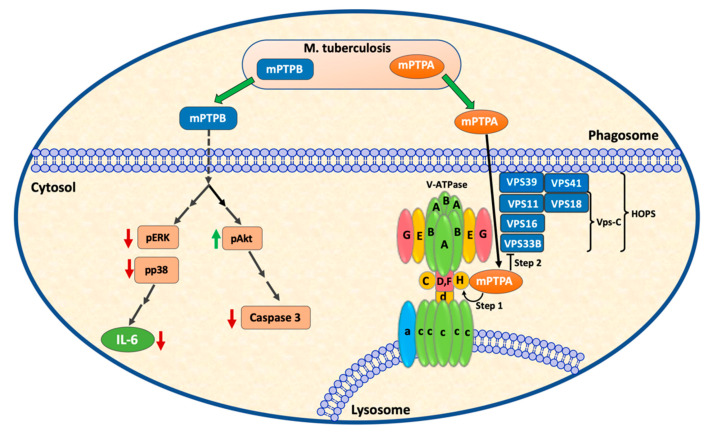
Roles of mPTPA and mPTPB in mediating pathogen−host interactions. mPTPA and mPTPB are secreted by Mtb during macrophage infection (shown by green arrows). mPTPA has been demonstrated to translocate to the macrophage cytosol. mPTPA binds to subunit H of V-ATPase (Step 1) to selectively localize to its catalytic substrate VPS33B at the phagosome–lysosome fusion interface. Dephosphorylation of VPS33B (Step 2) results in the exclusion of V-ATPase from the mycobacterial phagosome. mPTPB activity leads to increased Akt phosphorylation (green arrow) and decreased ERK1/2 and p38 phosphorylation (red arrows), resulting in decreased apoptotic activity and reduced production of IL-6 (red arrow), respectively.

Protein tyrosine phosphatases (PTPs) remove the phosphoryl group(s) from substrate proteins (Figure 1). Together with protein tyrosine kinases, PTPs regulate numerous cellular functions, such as cell growth, proliferation, differentiation, metabolism, and immune response. Not surprisingly, abnormal regulation of PTPs can cause cancer, diabetes and obesity, autoimmune disorders, and neurodegenerative diseases [4,5]. The importance of PTPs in human diseases is further emphasized by the observations that they are often utilized by microbial organisms to avoid host immune clearance [4]. Bacterial pathogens have developed diverse strategies to alter the host signaling processes to circumvent the hostile environment of the macrophages [6]. Studies have shown that Mtb encodes two PTPs, *Mycobacterium* protein tyrosine phosphatase A (mPTPA; Rv2234) and *Mycobacterium* protein tyrosine phosphatase B (mPTPB; Rv0153c), which are secreted by the bacterium into the cytoplasm of host macrophages. These phosphatases directly alter host signaling to evade the antimicrobial functions of the host (Figure 2), thereby promoting Mtb survival within the macrophages [7,8,9]. Consequently, mPTPA and mPTPB represent attractive targets for anti-TB drug development.

## 2. Role of Protein Tyrosine Phosphatases in *Mycobacterium Tuberculosis*

Macrophages provide the first line of immune defense against invading pathogens, including Mtb. Macrophages engulf every microorganism or foreign particle into phagosomes that consequently interact with the endocytic pathway, causing changes to membranes that allow the phagosome to recruit the vacuolar H+ATPase (V-ATPase) and hydrolases [10]. The presence of V-ATPase on the phagosome membrane creates an acidic compartment of pH 4.5–5.0, which indicates phagosome maturation. Acid activated hydrolases then mediate the killing of the invading microorganism or foreign particle. Available evidence suggests that mPTPA inhibits phagosome acidification and therefore prevents Mtb phagocytosis by the macrophage (Figure 2) [7,8]. Upon Mtb infection, macrophages also activate various host innate immune surveillance pathways. To avoid host immune clearance, mPTPB decreases the secretion of inflammatory cytokines and suppresses macrophage apoptosis (Figure 2) [9]. Indeed, both mPTPA and mPTPB are indispensable for Mtb intracellular survival.

### 2.1. Protein Tyrosine Phosphatase A (mPTPA)

**Figure 3:**
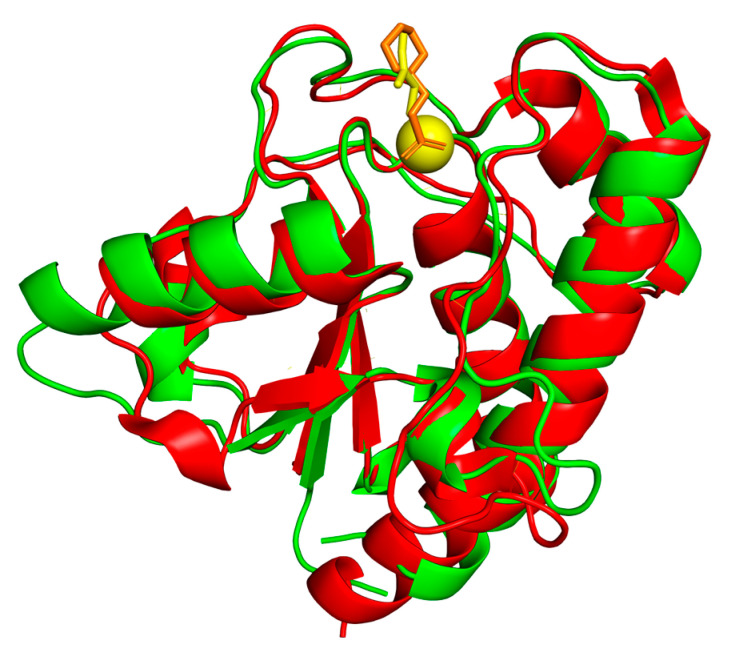
Comparison of the structures of mPTPA and the human LMW-PTPA. Ribbon diagram showing the superposition of mPTPA (red, PDB code: 1U2Q) and the human LMW-PTPA (green, PDB code: 5PNT). Chloride ion and glycerol molecule (shown in yellow) are shown in the active site of mPTPA, and the 2-(N-morpholino)-ethanesulfonic acid molecule (shown in orange) in the human LMW-PTPA.

mPTPA was first identified by Koul et al. from the genome sequence of Mtb H37Rv due to its homology with low molecular weight PTPs (LMW-PTPA) (Table 1) [11]. Similar to other PTPs, it contains the conserved C(X)_5_R(S/T) motif with the Cys11 serving as the nucleophile that attacks the phosphotyrosine residue on the substrate as shown in Figure 1 [12]. As shown in Figure 1, residues Cys11, Arg17, and Asp126 play a critical role in phosphatase activity of mPTPA. In addition to Cys11, a second cysteine (Cys16) is present in the active site of the enzyme and protects the Cys11 from oxidative inactivation by forming a reversible disulfide bond. The sequence comparison of mPTPA and LMW-PTPA shows conservation of residues in the DPYY loop of the active site and the overall 3D fold is similar, as shown in Figure 3. However, the surface of mPTPA shares little shape and sequence similarity with the LMW-PTPA. The differences in surface-charge distribution can be observed by analyzing the electrostatic surface diagrams of these proteins. The residues Trp48 and His49 in mPTPA are substituted by Tyr49 and Glu50 in LMW-PTPA, which creates a negatively charged patch near the opening of the active site. Moreover, the α4 region also showed significant differences due to the replacement of charged Asp98 and Lys102 in LMW-PTPA by the hydrophobic Leu96 and Leu100 in mPTPA. 

When secreted from Mtb into the host macrophage phagosomes, mPTPA disrupts the key components in the endocytic pathway, which results in the arrest of phagosome maturation [7,8]. Previous studies have shown that mycobacteria can evade the host’s immune system, causing phagosome acidification failure and phagosome maturation inhibition [13]. The homotypic fusion and vacuole protein sorting (HOPS) complex assembles on the phagosome membrane and is involved in activating lysosome fusion. Human vacuolar protein sorting 33B (VPS33B) is one of the members of this multi-protein HOPS complex C required for membrane trafficking and fusion. Bach et al. identified VPS33B as a cognate substrate of mPTPA by using electron microscopy on Mtb-infected macrophages (Figure 2) [7]. VPS33B is a class C vacuolar protein sorting complex (VPS-C), consisting of three subunits, VPS11, 16, and 18 [14]. mPTPA-mediated dephosphorylation of VPS33B inactivates the host protein complex VPS-C, leading to inhibition of phagosome–lysosome fusion [7]. It has been shown that binding of mPTPA to the V-ATPase is required for the dephosphorylation of VPS33B [8]. When mPTPA was deleted in Mtb, mycobacterial survival within human THP-1 macrophages was found to be impaired. These strains showed increased phagosome–lysosome fusion and transfer of lysosomal contents compared to the parental strain suggesting the importance of mPTPA in Mtb virulence [7]. Hence, the current understanding of phagosome maturation inhibition by mPTPA indicates a two-step process (Figure 2): (1) binding to V-ATPase, which brings mPTPA to the vicinity of its substrate; (2) dephosphorylation of VPS33B by mPTPA, which results in phagosome maturation arrest and inhibition of phagosome acidification. This blocks the phagosome–lysosome fusion in the infected macrophages, protecting the bacteria from destruction [13]. 

### 2.2. Protein Tyrosine Phosphatase B (mPTPB)

**Figure 4:**
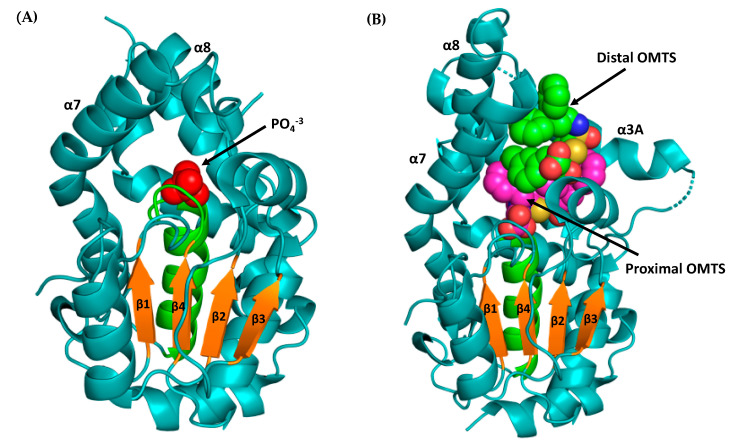
X-ray crystal structures of mPTPB. (**A**) X-ray structure of phosphate bound mPTPB in the flexible lid closed form (PDB code: 1YWF). Product phosphate shown in red. The flexible lid (α7-α8) is in closed form. (**B**) X-ray structure of OMTS ((oxalylamino-methylene)-thiophene sulphonamide) bound mPTPB in open form (PDB code: 2OZ5). Proximal OMTS shown in magenta and distal OMTS shown in green. The flexible lid (α7-α8) is in the open form to accommodate OMTS molecules. The p-loop and β-sheets (β1, β2, β3 and β4) are shown in green and orange, respectively.

mPTPB, a 30 kDa protein, shows an unusual fold, similar to the human myotubularin MTMR, that differs from the classic PTP fold (Table 1). The x-ray structure analysis of mPTPB revealed a flexible lid that covers the active site in closed phosphate (PO_4_^3−^) bound form but is open in an inhibitor (OMTS) bound form as shown in Figure 4 [15,16]. mPTPB displays a very large active site (unlike the narrow and deep active site pocket in classic PTPs) that is consistent with its phosphoinositide activity, in addition to its Tyr and Ser/Thr phosphatase activity [17]. Compared to the closed structure of the mPTPB-PO_4_ complex, the crystal structure of the mPTPB-OMTS ((oxalylamino-methylene)-thiophene sulphonamide) complex shows three significant structural variations. A large movement of the flexible lid (α7-α8) away from the catalytic pocket. Second, the disordered residues 85–104 in the mPTPB-PO_4_ structure formed a new α3A helix next to the active site pocket (Figure 4B). This formation of α3A helix also inverts the adjacent FPD loop that consists of the general acid Asp82 in PTPs. In the mPTPB-PO_4_ complex, the closed lid makes several intramolecular interactions with the rest of the protein, including hydrophobic interactions and salt bridges. 

It has been shown that Mtb lacking mPTPB cannot survive in macrophages and guinea pigs even though mPTPB is not required for Mtb growth in vitro [18]. Biochemical studies reveal that expression of mPTPB attenuates the MAPK and NF-kB pathways, leading to reduced production of inflammatory cytokines IL-1β and IL-6, factors that are important for upregulation of microbicidal activity, and initiates the immune response to Mtb infection [9,19]. Little effect on MAPK activity or IL-6 level was observed in cells expressing the catalytically inactive mPTPB/C106S. This result suggests that mPTPB phosphatase activity is essential for virulence. In addition to attenuation of the bactericidal responses mediated by IFN-γ, pathogenic mycobacteria also inhibit host cell apoptosis [9]. Programmed cell death of infected macrophages alerts the host immune system of foreign pathogen invasion. Thus, as a critical component of the innate immune defense system against Mtb, macrophage cell death is required to impede intracellular bacterial colonization. Evidence shows that mPTPB can also promote macrophage survival by augmenting Akt phosphorylation and suppressing caspase 3 activity [9]. Taken together, the results suggest that mPTPB acts to interfere with host defense mechanisms by blocking the bactericidal immune responses and increasing macrophage survival (Figure 2).

## 3. mPTPA Inhibitors

Due to their secretion into the macrophages, drugs targeting mPTPA and mPTPB do not need to be delivered across the poorly permeable waxy bacterial cell wall. In addition, potent and specific mPTPA and mPTPB inhibitors may have therapeutic value with a unique mechanism of action and speed up the tuberculosis treatment by allowing the macrophages to target the intracellular bacteria that remain after treatment with existing drugs. Waldmann et al. reported the first inhibitors for mPTPA by screening collections of a natural-product inspired and fragment-based library [20]. They identified several natural products with IC_50_ values ranging from 8.8 µM and 28.7 µM. However, all these natural products showed poor selectivity for mPTPA over other PTPs, such as VHR, Cdc25A, PTP1B, and CD45. Further efforts utilizing a fragment-based approach identified a novel hydroxypyrrol benzoic acid **1**, as a potent mPTPA inhibitor with K_i_ of 1.6 µM (Table 2). However, this analog showed poor selectivity (2-fold) against human PTPs such as PTP1B (K_i_ = 3.0 µM). 

Owing to its high structural similarity to pTyr, phosphonodifluoromethyl phenylalanine (F_2_Pmp) is one of the most commonly used nonhydrolyzable pTyr mimetics for PTP inhibitor development [21,22]. Ellman and co-workers reported on F_2_Pmp-based benzanilide derivative **2** with a K_i_ of 1.4 ± 0.3 µM for mPTPA (Table 2) [23]. When tested against a panel of human PTPs, **2** showed selectivity of 11-fold vs. the highly homologous HCPTPA, and 70-fold vs. mPTPB, PTP1B, TC-PTP, VHR, CD45 and LAR. 

Cefsulodin, a third-generation cephalosporin β-lactam antibiotic, was shown to exhibit inhibitory activity against several members of the PTP superfamily [24]. Fragmentation analysis of cefsulodin revealed α-sulfophenylacetic amide (SPAA) as a novel pTyr mimetic. Structure-guided optimization of SPAA led to compound **3**, with an IC_50_ of 160 nM for mPTPA (Table 2) [25]. Kinetic analysis revealed **3** as a reversible and competitive inhibitor of mPTPA (K_i_ = 56 ± 2.0 nM). The specificity of **3** for mPTPA was measured against mPTPB and a large number of mammalian PTPs, SHP1, SHP2, PTP1B, FAP1, LYP, TC-PTP, HePTP, Meg2, LAR, CD45, PTPα, PTPRG, VHR, VHX, Laforin, and Cdc14A, LMW-PTP. Compound **3** demonstrated high specificity for mPTPA, displaying >20-fold potency over all the PTPs tested.

## 4. mPTPB Inhibitors

The absence of human orthologues of mPTPB makes it a highly attractive therapeutic target for TB drug discovery because minimal side effects are expected for the host. Over the last 15 years, numerous small molecule mPTPB inhibitors have been disclosed. To identify novel natural product-derived phosphatase inhibitors, Waldmann and co-workers screened a set of chemically diverse natural products against VE-PTP, SHP2, PTP1B, mPTPA, mPTPB, Cdc25A and VHR [26]. The screen led to the discovery of four unprecedented inhibitor classes for SHP2, PTP1B, VE-PTP and mPTPB with high hit rates. The most potent inhibitor identified for mPTPB was **4**, with an IC_50_ of 0.36 µM (Table 3). Importantly, this inhibitor showed over 100-fold selectivity in comparison with a panel of PTPs tested. Other studies on natural products and their derivatives for mPTPB inhibition have had limited success due to high molecular weight, moderate potency and poor selectivities [27,28,29]. 

**Figure 5:**
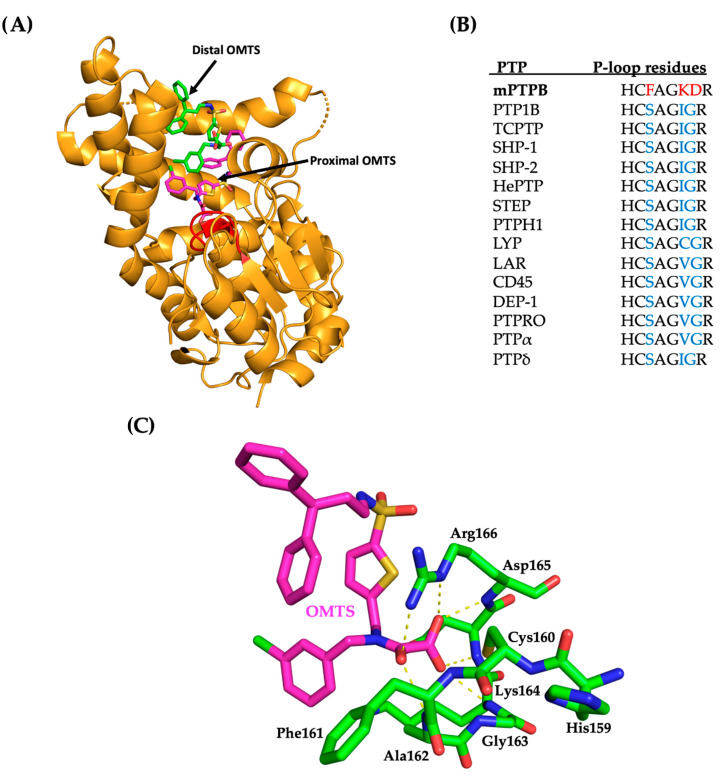
(**A**) The complex of mPTPB and OMTS (pdb code: 2OZ5) is shown in orange. The proximal and distal molecules of OMTS are shown in magenta and green, respectively. P-loop residues 159HCFAGKDR166 are shown in red. (**B**) A comparison of P-loop residues of mPTPB and other members of the PTP family. The residues in the P-loop that make contacts to OMTS are shown in red and are unique in mPTPB compared to other PTPs (shown in blue color). (**C**) Molecular interactions between proximal OMTS (in magenta) and P-loop residues (in green) in mPTPB (pdb code: 2OZ5). Hydrogen bonding interactions shown as yellow dotted lines.

A library screen of several oxamic acid derivatives resulted in (oxalylamino-methylene)-thiophene sulfonamide (OMTS) **5**, as an inhibitor for mPTPB with an IC_50_ of 0.44 ± 0.05 μM and > 60-fold specificity for mPTPB over six human PTPs (Table 3) [15]. The mode of inhibition studies revealed OMTS as a classic competitive inhibitor with an apparent K_i_ value of 0.33 ± 0.04 μM. The x-ray structure of the mPTPB complex with the OMTS revealed the binding of two molecules of OMTS, one in the active site and another one in the nearby secondary binding pocket (Figure 5A). The residues (F161, K164 and D165) in the P-loop that are making interactions with OMTS are unique in mPTPB compared to many other human PTPs (Figure 5B). The P-loop F161 residue in mPTPB makes hydrophobic interactions with OMTS, whereas human PTPs SHP-1, SHP-2, PTP1B, LAR, and LYP have a serine residue in the same position as shown in Figure 5B. Similarly, K164 (I or V or C in human PTPs) and D165 (G in human PTPs) represent non-conserved residues compared to human PTPs. 

He et al. developed a multicomponent Mannich type reaction between pyrrole, formaldehyde, and aniline to generate several salicylic acid-based inhibitors for mPTPB [30]. This strategy resulted in inhibitor **6** with an IC_50_ of 1.5 ± 0.2 μM and >50-fold specificity vs. PTPs tested (Table 3). He et al. also developed a novel, double click chemistry strategy for the acquisition of potent mPTPB inhibitors [31]. The strategy was to increase the interactions with peripheral pockets surrounding the active site of mPTPB by incorporating two alkyne handles into the central core structure, which would allow it to react with two azides, thus creating a tridentate molecule. The most potent inhibitor obtained from this approach was **7** (Table 3), with an IC_50_ value of 160 nM and over 25-fold specificity for mPTPB over other PTPs. 

Using a diversity-oriented synthesis (DOS) strategy, He et al. synthesized a series of bicyclic salicylic acid-based inhibitors for mPTPB [32]. They identified aminothiazole salicylic acid derivative **8** as an inhibitor for mPTPB with an IC_50_ of 2 μM and >20-fold specificity over other human PTPs (Table 3). The bicyclic salicylic acids generated by the DOS strategy provide a rich source of promising starting points for developing inhibitors targeting members of the PTP family. Isoxazole carboxylic acids are commonly used as pTyr mimetics for PTP inhibition and have been successfully used to generate bi-dentate inhibitors for PTP1B [33]. Ellman’s group conducted a fragment-based substrate activity screening method for the identification of mPTPB inhibitors [34]. This method yielded isoxazole inhibitor **9** with a K_i_ of 220 nM (Table 3). In addition, inhibitor **9** showed good selectivity against a panel of mycobacterial and human PTPs, such as mPTPA, VHR, TC-PTP, CD45 and LAR.

An indole salicylic acid-based focused library strategy by Zeng and co-workers generated a 3-iodo indole salicylic acid core with a K_i_ of 1.2 ± 0.1 μM [35]. The replacement of 3-iodo with various amide containing linkers provided compounds with the highest potency and selectivity due to their binding to peripheral binding pockets near the active site. Compound **10** obtained by modification of the side chain showed an IC_50_ of 0.079 ± 0.010 μM. Furthermore, **10** exhibited at least 100-fold selectivity for mPTPB over several other 19 PTPs, including mPTPA and PTP1B. The treatment of mPTPB expressing Raw264.7 macrophages with **10** restored the IFN-γ induced activation of ERK1/2 in a concentration-dependent manner. Moreover, **10** also normalized AKT activity in mPTPB containing cells to the same extent as the vector cells.

Zhang’s group identified inhibitor **11,** with excellent cellular activity, from a combinatorial library of benzofuran salicylic acid derivatives synthesized by click chemistry [9]. It was also shown that mPTPB inhibition with **11** in macrophages reverses the immune responses induced by the bacterial phosphatase in the host. The results provided the necessary proof-of-concept data to support the belief that potent inhibitors of the mPTPB may serve as anti-TB agents. Later, the same group developed another inhibitor from the same precursor used for the development of **11** [36]. Medicinal chemistry-oriented optimization of the benzofuran salicylic acid precursor resulted in a highly potent (IC_50_ = 38 nM) and selective inhibitor, **12**. Importantly, **12** is also capable of reversing the altered host immune responses and restoring the macrophage’s full capacity to secrete IL-6 and undergo apoptosis in response to INF-γ stimulation. The study further demonstrated the utility of bicyclic salicylic acid pharmacophores as highly successful cores for the development of PTP inhibitors. 

As discussed above, cefsulodin, an FDA-approved drug serves as a moderately potent inhibitor for various PTPs due to the presence of a SPAA core. Cefsulodin also shows moderately potent mPTPB inhibition with an IC_50_ of 16 μM [37]. Fragmentation analysis revealed that the SPAA pharmacophore is a weak inhibitor of mPTPB with an IC_50_ of 180 μM. Fragment-based optimization of SPAA led to the discovery of several compounds with excellent inhibitory activity for mPTPB. Among them, compound **13** demonstrated an IC_50_ of 18 nM (Ki of 7.9 nM) and with over 10,000-fold selectivity against 25 phosphatases. It also shows excellent activity in blocking mPTPB’s function in macrophages. 

Tabernero and co-workers reported isoxazole-3-carboxylic acid derivatives as inhibitors of mPTPB [38]. Their efforts led to the discovery of an orally bioavailable inhibitor, **15**, with an IC_50_ of 2.8 μM. Though compound **15** was 7-fold less potent than their most potent compound **14** (IC_50_ of 0.4 μM), **15** demonstrated improved pharmacological properties, oral bioavailability and showed an excellent pharmacokinetic profile. Therefore, **15** was selected for evaluation of cellular activity and efficacy studies in animal models. Inhibitor **15** showed efficacy in decreasing the bacterial burden in a guinea pig model of infection and improvement in the spleen and lungs’ pathology. This is consistent with Chauhan et al.’s observation of protection at the primary site of infection in guinea pigs when the mPTPB gene is mutated [39]. 

More recently, Ruddraraju et al. identified N-phenyl oxamic acid (IC_50_ = 5.4 μM for mPTPB and >20-fold selectivity over PTP1B and SHP2) as a potent minimal unit for mPTPB inhibition [40]. Further structure-guided medicinal chemistry efforts of the initial hit **16** (IC_50_ = 0.257 μM) resulted in several tight binding compounds with <10 nM K_i_ and ~4500-fold selectivity against a panel of 25 PTPs. Among them, compound **15** showed superior selectivity, solubility and cell permeability, which led to its selection for biological studies. Kinetic, molecular docking and site-directed mutagenesis studies revealed them as active site-directed reversible inhibitors of mPTPB. Compound **17** showed a decrease in AKT and ERK1/2 phosphorylation and an increase in p38 phosphorylation in Raw267.4 mouse macrophages. These oxamic acid inhibitors are the most potent in vitro inhibitors of mPTPB described to date. High potency, exceptional selectivity, low molecular weight and excellent pharmacological properties make these inhibitors excellent candidates for therapeutic development.

## 5. Perspectives and Future Directions

TB is an infectious disease caused by the Mtb bacteria and usually attacks the lungs. The complex nature and lengthy duration of current therapy and the resulting emergence of MDR and XDR TB support the need for novel therapies for the treatment of TB. As key secreted virulence factors for Mtb survival within host macrophages, mPTPA and mPTPB have garnered substantial interest as novel anti-TB targets. These phosphatases that interfere with innate host defense mechanisms are required for optimal bacillary survival of Mtb within macrophages and in animal models. Targeting mPTPA and mPTPB for the treatment of TB offers an alternative strategy to the traditional antibiotic approaches and could provide therapeutic agents that overcome the antibiotic resistance. In practice, the success of a small molecule-based drug discovery project depends on the availability of potent and specific inhibitors with drug-like properties. As discussed above, significant advances were made in the mPTPB inhibitor development, and, to a lesser extent for mPTPA. Perhaps the absence of a human ortholog of mPTPB prompted researchers to focus on drug discovery efforts. Recently, Ghattas and co-workers conducted a druggability assessment on 17 PTPs [41]. They characterized and assessed the active sites of these PTPs for their ability to bind drug-like molecules. Their assessment revealed that only two of 17 PTPs, namely mPTPB and GLEPP-1, are likely druggable due to their large hydrophobic active sites. This is consistent with the successful discovery of several highly potent drug-like small molecules for mPTPB. For instance, salicylic acid derivative **11**, sulfonic acid derivative **12**, and oxamic acid inhibitor **16,** discussed above, possess low molecular weight, nanomolar inhibition with >1,000-fold selectivity and excellent drug-like properties. 

As discussed above, the secretory PTPs target multiple pathways within the host. Thus, it is important to identify if there are any conserved host proteins that are dephosphorylated by phosphatases from different bacteria. This allows the researchers to design broad-spectrum inhibitors that can work against different pathogens in which phosphatases play a critical role, such as *M. tuberculosis, Yersinia S. typhi, and S. aureus*. Mycobacterial phosphatases have emerged as key players in blocking phagosome acidification (mPTPA) and in interfering with the MAPK and AKT signal transduction pathways (mPTPB). Targeting these virulence factors from *Mycobacterium tuberculosis* to restore host signaling pathways could be an effective alternative approach to traditional antibiotics. Given the novel modes of action, new animal models that better recapitulate human immune reactions upon Mtb infection are greatly needed in order to assess the therapeutic potential of targeting the virulent Mtb PTPs. To that end, mPTPA inhibitor **3** and mPTPB inhibitor **12** augment the bactericidal activity of the standard antitubercular regimen of isoniazid-rifampicin-pyrazinamide (HRZ) in a guinea pig model of chronic TB infection [25]. The inhibitors discussed above are not only useful as starting points for the development of therapeutic agents for TB but can also serve as molecular probes in understanding the structure and function of these enzymes in TB pathogenesis. 

## Figures and Tables

**Table 1:** Comparison table for mPTPA and mPTPB.

Characteristic	mPTPA	mPTPB
Molecular Weight	17.5 kDa (163 aa)	30 kDa (293 aa)
Catalytic Residue	Cysteine 11	Cysteine 160
P-loop residues	11-CTGNICRS	160-CFAGKDRT
Specificity	pTyr specific	Triple specificity (pTyr, pSer/pThr and phospholipid)
X-ray structures (pdb)	1U2P, 1U2Q	1YWF, 2OZ5
Substrates	VPS33B	Unknown
Role during Infection	Inhibits phagosome acidification and maturation; blocks V-ATPase recruitment to phagosome	Activates Akt; inhibits p38 and ERK1/2 signaling pathways. Inhibits IL-6 production and caspase 3 activation.
Phosphorylation	PtkA and STPKs	No
Reaction with H_2_O_2_	Leads to oxidative inactivation	Stable compared to mPTPA
S-nitrosylation	Yes, at Cys53 by Nitric oxide	No, protected by lid domain

Aa = amino acids, kDa = kilodaltons.

**Table 2:** Chemical structures and IC_50_ values of various mPTPA inhibitors.

Entry	Structure	mPTPA IC_50_ (µM)	mPTPB IC_50_ (µM)	Selectivity (Ref.)
**1**	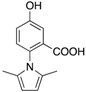	1.6 ± 0.4 (Ki)	NA	2-fold for hPTP1B (20)
**2**	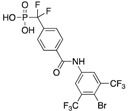	1.4 ± 0.3 (Ki)	>100 (Ki)	>70-fold vs. PTP1B, Tc-PTP, VHR, CD45 and LAR; 11-fold vs. HCPTPA (23)
**3**	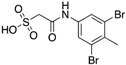	0.160 0.056 ± 0.002 (Ki)	3.2	>20-fold vs. a panel of 17 human PTPs (25)

NA = not reported. Ref. = Reference.

**Table 3:** Chemical structures and IC_50_ values of various mPTPB inhibitors.

Entry	Structure	mPTPB IC50 (µM)	mPTPA IC50 (µM)	Selectivity (Ref.)
**4**	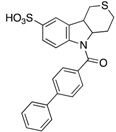	0.36 ± 0.12	>100	>100-fold vs. VE-PTP, SHP2, PTp1B, CDC25A and VHR (26)
**5**	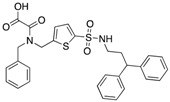	0.44 ± 0.05 (Ki)	NA	>60-fold vs. a panel of six human PTPs (15)
**6**	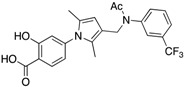	1.5 ± 0.2	180 ± 30	>50-fold vs. a panel of 5 PTPs (30)
**7**	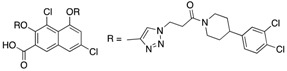	0.160 ± 0.010	7.8	>25-fold vs. a panel of 17 human PTPs (31)
**8**	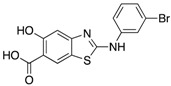	2.0 ± 0.1	NA	>20-fold vs. a panel of 8 PTPs (32)
**9**	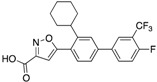	0.22 (Ki)	>50	225-fold vs. VHR and TC-PTP; 35-fold vs. CD45; 98-fold vs. LAR (33)
**10**	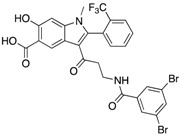	0.079 ± 0.01 0.050 (Ki)	7.8 ± 0.5	>100- fold vs. 19 PTPs (35)
**11**	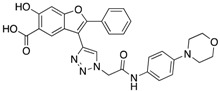	1.26 ± 0.22	77.3 ± 5.1	>11-fold vs. 12 PTPs (9)
**12**	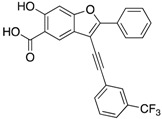	0.038 ± 0.002	2.5	>37-fold vs. a panel of 17 PTPs (36)
**13**	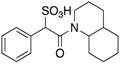	0.018 ± 0.002 0.0079 (Ki)	>200	>10,000-fold vs. 24 PTPs (37)
**14**	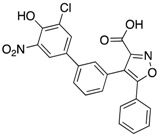	0.40 ± 0.05	30.2 ± 1.4	>750-fold vs. PTP1B and >32-fold vs. VHR (38)
**15**	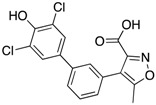	2.98	NA	NA (38)
**16**	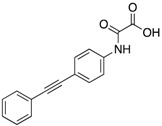	0.257 ± 0.008	NA	>390-fold vs. PTP1B and SHP2 (40)
**17**	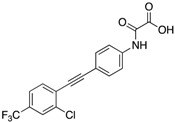	0.0064 ± 0.0005 0.0027 (Ki)	>30	>4500-fold vs. 25 PTPs (40)

NA = not reported. Ref. = Reference.
